# DNA damage response priming by CK2: a matter of life or cell death in root apical meristems

**DOI:** 10.1093/plcell/koab018

**Published:** 2021-01-25

**Authors:** Valentin Hammoudi

**Affiliations:** Institute of Biology, Applied Genetics, Freie Universit�t Berlin, Albrecht Thaler Weg 6, 14195 Berlin, Germany

Aluminum (Al) is a major constituent of soils and takes the form of Al^3+^ ions in acidic soils. Besides impeding cell elongation, internalized Al^3+^ inhibits DNA replication and causes DNA fragmentation. Consequently, root growth becomes largely impaired (Sjogren et�al., 2015). In agriculture, Al toxicity frequently goes hand in hand with phosphorus (P)-limitation: P is chelated by Al^3+^, reducing the bioavailability in soils of inorganic phosphate (Pi) to plants. Accordingly, Pi deficiency symptoms resemble to some degree those of Al toxicity, including overall root stunting, cell wall stiffening, callose accumulation, and loss of division competence in root meristem cells, a process known as meristem exhaustion.

Using a large chemical screen, Wei et�al. (2021) searched for chemical compounds that abolish the root meristem exhaustion triggered by Al toxicity in Arabidopsis. The authors report on the identification of a small chemical called C43, which inhibits Casein Kinase 2 (CK2), a pleiotropic and widely conserved protein kinase in eukaryotes. They use both C43 and the *cka123* mutant, a knockdown line for *CK2*, to investigate the role of CK2 in response to Al toxicity.

Involvement of CK2 in the DNA damage response (DDR) pathway had previously been suggested by the hypersensitivity of *cka123* mutants upon DNA damage (Moreno-Romero et�al., 2012). In response to genotoxic stress, the DDR is orchestrated by the transcription factor SUPPRESSOR OF GAMMA RESPONSE 1 (SOG1), a master regulator of DDR in plants and functional homolog of the mammalian p53. The massive transcriptional reprogramming triggered by SOG1 results in cell cycle arrest, DNA repair, and in some cases also programmed cell death (Bourbousse et�al., 2018; Ogita et al., 2018). Interestingly, similar to C43-treated plants and *cka123* mutants, loss-of-function *sog1* mutants fail to arrest root growth in response to Al (Sjogren et�al., 2015), supporting the idea that both CK2 and SOG1 act in the same pathway. In line with this, no additional increase in root growth in the presence of Al could be observed in C43-treated *sog1* mutant compared to wild-type plants, indicating that CK2 likely operates upstream of SOG1.

SOG1 is activated by phosphorylation: upon zeocin-induced double-strand breaks, five SQ motifs in SOG1 transcription regulatory domain were previously identified as phosphorylated by the kinase ATAXIA-TELANGIECTASIA MUTATED (ATM), with phosphorylation being required for SOG1 function (Yoshiyama et�al., 2013). With proteomics, Wei et�al. (2021) identified in the same domain a new phosphorylation site (T423) that corresponds to a CK2 phosphorylation motif conserved in dicots. Subsequently, the Al insensitivity of *sog1* observed with root growth was not fully abolished by reintroducing SOG1 with the T423A mutation, implying decreased SOG1 activity. Additionally, not only did the C43-mediated inhibition of CK2 reduce the transcriptional induction of some SOG1-target genes, but also it strongly attenuated ATM-dependent phosphorylation of SOG1. Upon DNA damage, the activation of SOG1 by ATM appears to be primed by CK2-dependent phosphorylation, although a direct inhibition of ATM activity by C43 cannot be totally ruled out.

Since responses to Al toxicity and low Pi availability potentially converge on Al tolerance factors, C43 was also used to investigate a potential involvement of CK2 in response to P starvation. The loss of CK2 activity resulted in an increased Pi uptake and a failed activation of SOG1. Strikingly, an attenuated DDR predominantly contributed to the rescue of the inhibited root growth phenotype upon Pi limitation, attributing to SOG1 a pivotal role in the response to root growth to low Pi availability in soil.

**Figure:**
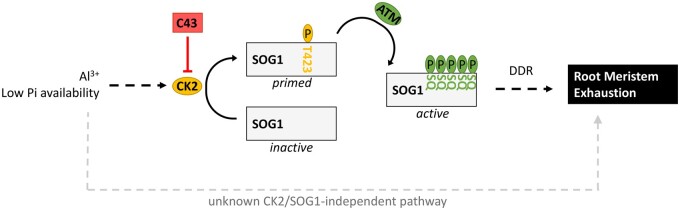
Proposed model for signaling in response to Al toxicity and Phosphate starvation. In Al toxicity or low Pi conditions, CK2 likely directly phosphorylates and primes SOG1 for the activation of the DDR, which results in root meristem exhaustion. Wei et�al. (2021) show that C43, an inhibitor of CK2, prevents SOG1 from being primed, which leads to tolerance toward Al toxicity and P starvation. Circled Ps represent phosphorate groups; gray arrows represent a potential CK2- and/or SOG1-independent pathway contributing to root meristem exhaustion in response to Al toxicity and P starvation; dashed arrows represent signaling that was not studied by Wei et�al. (2021). (*Figure credit V. Hammoudi*).

By shedding light on the close relationship between DDR, CK2, and signaling responses to Al toxicity and P starvation, the work of Wei et�al. (2021) brings into question the identification of the factors responsible for meristem exhaustion downstream of SOG1. In addition, the root growth recovery in both C43-treated plants and in *sog1* mutants is only partial, suggesting that other factors that do not belong to SOG1 pathway also probably contribute to the response to these two stresses. The potential involvement of these other factors in response to Al toxicity and P starvation should be addressed in future studies to further lift the veil on the process of meristem exhaustion. 
